# Slow Wave Activity During Nocturnal Sleep in Hypersomnolence Disorder

**DOI:** 10.1111/jsr.70353

**Published:** 2026-05-29

**Authors:** Jesse D. Cook, Brady A. Riedner, Meredith E. Rumble, Michael R. Goldstein, Ana Maria Vascan, Kieulinh M. Tran, David T. Plante

**Affiliations:** ^1^ Department of Psychiatry University of Wisconsin School of Medicine and Public Health Madison Wisconsin USA; ^2^ Department of Psychology University of Wisconsin Madison Wisconsin USA; ^3^ Department of Kinesiology, School of Education University of Wisconsin Madison Wisconsin USA; ^4^ Department of Psychiatry Beth Israel Deaconess Medical Center & Harvard Medical School Boston Massachusetts USA

**Keywords:** hypersomnia, hypersomnolence, hypersomnolence disorder, sleepiness, slow wave activity, slow waves

## Abstract

Slow waves during non‐rapid eye movement (NREM) sleep are associated with the restorative aspects of sleep. Previous research has suggested reduced normalized slow wave activity (SWA), particularly in the first cycle of NREM sleep, as a feature of hypersomnolence disorder (HD); however, the scarcity of existing research highlights the need for replication studies. Thus, the aim of this investigation was to evaluate normalized SWA in patients with HD, relative to healthy sleeper controls (HSC). Nocturnal polysomnography from 37 unmedicated HD clinical patients were compared against 29 HSC. Six‐channel electroencephalographic data from polysomnography was processed according to consensus standards. Normalized SWA (0.5–4.5hz) was calculated from artefact‐free NREM epochs for all‐night data, as well as sleep cycle 1, 2, and 3. Normalized SWA was compared across groups, with adjusted analyses accounting for age, sex, body mass index, and depressive symptomatology. Post hoc analyses compared HD who met objective criteria for idiopathic hypersomnia (HD/IH+), those who did not (HD/IH−), and HSC. HD demonstrated significantly reduced all‐night, normalized SWA in unadjusted analyses across all channels, without significant differences observed in fully adjusted analyses. In cycle 1, HD displayed significantly reduced normalized SWA across all channels in both unadjusted and adjusted analyses. HD/IH+ and HD/IH− were statistically comparable in normalized SWA across all analyses. This study provides further evidence of reduced SWA, particularly during cycle 1 of NREM sleep, as a core feature of HD. Future studies that investigate the causal role of slow waves in the pathophysiology of HD are warranted.

## 1 Introduction

Unexplained hypersomnolence, defined as excessive daytime sleepiness with normal to prolonged sleep duration, is a common, but understudied issue in sleep medicine (Ohayon et al. 2012; Plante et al. 2024). While hypersomnolence is often explained by an existing medical, sleep, or circadian rhythm disorder, many patients experience hypersomnolence without an overarching explanation (Pérez‐Carbonell et al. 2022). The Diagnostic and Statistical Manual of Mental Disorder, 5th Edition uses hypersomnolence disorder (HD) to classify unexplained hypersomnolence (American Psychiatric Association 2013). HD is quite common, affecting roughly 1.5% of the population (Ohayon et al. 2012), however, it is poorly understood, with an unknown pathophysiology and no established biomarkers (Cook et al. 2019; Trotti and Arnulf 2021; Nevsimalova et al. 2021).

Treatment options for HD are primarily pharmacological and focused on symptom management rather than disease modification. At present, there are no curative treatment options, and many patients experience residual symptoms despite utilizing frontline options, and some patients abandon medication use completely, potentially due to the ineffectiveness of treatment or intolerability of side effects. Identifying biological signatures of HD is a critical step towards novel, sustainable, and efficacious interventional strategies. Biomarkers can provide insight into the underlying pathophysiology, which can guide future research with targeted therapeutics.

Slow waves are high amplitude, low frequency rhythmic electroencephalographic (EEG) oscillations that predominate non‐rapid eye movement (NREM) sleep, exist as the hallmark of N3 (“Slow wave sleep”; SWS), and are recognized as a valid and reliable indicator of sleep intensity and depth (Dijk 2009; Fehér et al. 2021). These EEG oscillations arise from synchronized fluctuations in cortical and thalamic neurons, as they alternate between depolarized “up‐states” and hyperpolarized “down‐states.” (Bersagliere and Achermann 2010; Timofeev and Chauvette 2011) Slow waves play critical roles in cortical development and maturation (Bersagliere and Achermann 2010), as well as the regulation of important biological processes (Klinzing et al. 2019; Tononi and Cirelli 2006; Besedovsky et al. 2019; Wisor et al. 2013), while also being tightly connected to sleep homeostasis and the restorative aspects of sleep (Dijk 2009; McCarter et al. 2022). Given that nonrestorative sleep is a cardinal feature of HD, it is logical to hypothesize that slow wave abnormalities may contribute to the development and severity of HD.

Slow wave activity (SWA) is commonly utilized to quantify slow waves during sleep. SWA (μV/Hz) captures the spectral power across the delta band (~0.5–4.5hz), which spans the oscillatory range that defines slow waves. Research has established SWA as a marker of sleep homeostasis and sleep need. This relationship is most saliently evidenced by increased SWA during recovery sleep following periods of prolonged wakefulness when homeostatic need for sleep is high (Dijk 2009; Mander et al. 2010; Achermann et al. 2017). Given the role of SWA in sleep restoration, a potential hypothesis is that reductions in SWA contribute to the nonrestorative sleep in HD. Recently, our laboratory explored SWA in HD using high‐density EEG, demonstrating centroparietal SWA reductions in HD, relative to healthy sleeper controls (Plante et al. 2019). The reductions were most prominent in the first cycle of NREM sleep. However, replication of this finding in a separate sample is necessary to establish reduced SWA as a marker of HD. Furthermore, SWA reductions in HD identified using a standard clinical six‐channel EEG sleep montage are required to increase the generalizability of findings.

As such, this investigation examined differences in SWA between unmedicated, well‐characterized patients meeting diagnostic criteria for HD against a sample of healthy controls without sleep complaints (HSC). Based on the available data, we hypothesized that HD would display reduced SWA relative to HSC, with the most pronounced reductions in central EEG derivations during the first cycle of NREM sleep.

## 2 Methods

### 2.1 Data, Ethics Approval, and Sample Formation

Unmedicated patients referred for polysomnography and multiple sleep latency testing for suspected CNS hypersomnolence disorder at Wisconsin Sleep, the sleep center affiliated with of the University of Wisconsin‐Madison, were recruited from 2016 to 2022 for this study. Healthy sleeper comparison (HSC) participants were recruited from the greater Madison, WI community and completed identical study procedures as hypersomnolent patients. All participants were monetarily compensated for their participation and study procedures were approved by the UW‐Madison Health Sciences Institutional Review Board.

Inclusion criteria for all participants included age (18–65 years old), absence of polysomnographic evidence of a primary sleep disorder that could cause hypersomnolence (i.e., obstructive sleep apnea; narcolepsy), reported habitual sleep duration < 7 h per night, history of significant head trauma, current or history of significant neurologic or medical illness (e.g., seizure disorder, heart failure), nicotine use > 15 cigarettes per day, consumption of > 3 caffeinated beverages per day, imminent risk for self‐harm or suicide. Additionally, women who were currently pregnant, < 6 months post‐partum, or planning to become pregnant during the study were excluded.

HSC needed to be absent of clinically significant subjective daytime sleepiness (ESS ≤ 10) (Johns 1991), view themselves as healthy sleepers, and report no difficulty staying awake, no desire to sleep during the day, and a habitual sleep duration between 7 and 9 h. HD participants met criteria for hypersomnolence disorder (American Psychiatric Association 2013), verified by chart review by a physician board‐certified in sleep medicine and psychiatry (DTP).

### 2.2 Collected Measures

#### 2.2.1 Demographics, Depressive Symptom Severity, and Self‐Report Measures of Hypersomnolence

All participants provided age, biological sex at birth, race, ethnicity, and height and weight data to calculate body mass index (BMI; kg/m^2^). Participants completed the inventory of depressive symptomatology—self‐report (IDS‐SR) to capture depressive symptom severity (Rush et al. 1996). Hypersomnolence‐specific self‐report measures were also completed, including the Epworth sleepiness scale (ESS) (Johns 1991), hypersomnia severity index (HSI) (Kaplan et al. 2019), sleep inertia questionnaire (SIQ) (Kanady and Harvey 2015), and functional outcomes of sleepiness scale—10 item (FOSQ‐10) (Chasens et al. 2009). Total scores for the IDS‐SR, ESS, HSI, and FOSQ‐10 were computed, as well as a symptom specific score from the HSI (HSI—Symptoms), which is formulated by the summation of the first four items. Higher values on these metrics reflect greater symptom severity, except for the FOSQ‐10 where lower values reflect greater symptom severity.

#### 2.2.2 Polysomnography and Objective Measures of Hypersomnolence

Participants underwent a single night of ad libitum, overnight in‐laboratory polysomnography (PSG). Participants arrived at the center during the early evening of their PSG evaluation. They were fitted with standard PSG equipment (Berry et al. 2015). Bedtime was determined to align with their habitual sleep schedule and participants were not awoken at a prescribed time while remaining minimally disturbed throughout the duration of the evaluation. PSG included six‐channel electroencephalography (EEG; F3, F4, C3, C4, O1, O2), electrooculogram, sub‐mental electromyogram, electrocardiogram, bilateral tibial electromyogram, respiratory inductance plethysmography, pulse oximetry, and a position sensor. Sleep was scored in real‐time by a trained technologist in 30‐s epochs according to American Academy of Sleep Medicine (AASM) criteria (Berry et al. 2015). All PSG data were collected using Compumedics Profusion (Compumedics, Victoria, Australia) software and hardware.

Following the PSG, participants underwent a daytime multiple sleep latency test (MSLT) in accordance with AASM clinical guidelines (Littner et al. 2005). Mean sleep latency (MSL) across all available nap opportunities served as the primary outcome from the MSLT. The study design interleaved two study time points between naps across the MSLT protocol, with participants completing additional objective measures of hypersomnolence at each of these two study timepoints: Pupillographic sleepiness test (PST) (Wilhelm et al. 1998) and 10‐min version of the psychomotor vigilance task (PVT) (Thomann et al. 2014; Plante et al. 2020). For the PST, the pupillary unrest index served as the primary outcome, while median reaction time (medRT) and number of lapses after Tukey transformation (Lapses) served as outcomes from the PVT, with a lapse defined by a response > 500 milliseconds. Outcomes from the PST and PVT were averaged across both timepoints for each participant.

### 2.3 EEG Processing, Artefact Detection, and Slow Wave Activity Computation

A standardized EEG processing pipeline, consistent with consensus sleep EEG methodological guidelines, was applied to participant EEG data from the six available channels (F3, F4, C3, C4, O1, and O2) (Cox and Fell 2020; Mariani et al. 2018; Purcell et al. 2017). Average mastoid referenced time‐series EEG data for each channel was downsampled to 128hz, first‐order high‐pass (0.1 hz) filtered (2‐way least‐squares FIR), and band‐pass filtered (0.3 to 30hz). Time‐series data for each channel were converted to the frequency domain using the approach described by Mariani et al. (2018) (Mariani et al. 2018). Specifically, power spectral density (PSD) was computed using Welch's method, using 10 overlapping 4‐s sub‐epochs for each 30‐s epoch, with a 50% tapered cosine (Tukey) window. The MATLAB function *pwelch* was used for PSD calculation (The MathWorks Inc. 2024).

Artifactual epoch detection was then performed using a combination of automatic and manual approaches. Manual detection was performed by the same person (JDC), and occurred blinded to participant group. Initially, raw time‐series signals were visualized to examine signal integrity across the night. Then, automatic detection methodology employed in the National Sleep Research sleep EEG processing pipeline was applied on each channel independently to identify bad epochs (Mariani et al. 2018; Purcell et al. 2017). This process employed Hjorth parameter evaluation on time‐series data, followed by Bueckelmuller threshold detection on PSD data (performed three times iteratively). Cleaned power spectra were visually inspected to ensure quality of cleaning. The multipronged cleaning approach resulted in channel‐specific artifactual epochs for each participant, along with a small subset of channels removed from the dataset due to inadequate signal quality. To account for interindividual variation that exists in EEG power (often due to non‐pathological factors), normalized slow wave activity (SWA; 0–5–4.5 hz) was computed for each channel on data from artefact‐free N2 and N3 staged epochs, in alignment with traditionally utilized methodology (Wilckens et al. 2016; Davidson et al. 2025). For each channel, absolute power was summed across bins corresponding to the SWA band, divided by the total summed power across all bins in that channel, and multiplied by 100 (i.e., proportion of total power accounted for by the SWA band).

Sleep cycles were computed using the *sleep_cycles* function for MATLAB, open access through the National Sleep Research Resource Github repository (https://github.com/nsrr/SpectralTrainFig). Normalized SWA was computed from artefact‐free N2 and N3 staged epochs for sleep cycles 1, 2, and 3 based on the cycle start and end points derived from *sleep_cycles*.

### 2.4 Statistical Analyses

Group comparisons between HD and HSC were performed across available sample characteristics, with linear regression utilized for continuous characteristics (age, BMI, and IDS‐SR) and chi‐square test of independence for categorical characteristics (sex, race, and ethnicity). Descriptive values are reported for each group along with the *p* value from the statistical comparisons.

Group differences between HD and HSC across self‐report and objective hypersomnolence measures, polysomnography variables, and SWA were evaluated using linear regression, with unadjusted and adjusted analyses performed. All adjusted regressions included age, sex, BMI, and IDS‐SR as covariates. Group means ± standard deviation are presented for each outcome. The regression coefficient (*β*), standard error (SE), *p* value, and partial eta‐squared ($\eta_{p}^{2}$) are reported from all unadjusted and adjusted analyses. For SWA, analyses were performed on all‐night data, as well as data from sleep cycle 1, 2, and 3.

After completing the primary analyses, post hoc analyses were performed that explored differences between HD participants who met the objective criteria for idiopathic hypersomnia (HD/IH+), those who did not (HD/IH−), and HSC. The PSG (≥ 660 min on ad libitum polysomnography) and MSLT (≤ 8 min) criteria outlined in the International Classification of Sleep Disorders, 3rd Edition, Text Revision (American Academy of Sleep Medicine 2023) were used to stratify the HD group into HD/IH+ and HD/IH−. Comparisons were performed across hypersomnolence measures, polysomnography, and SWA. For these analyses, Analysis of Covariance (ANCOVA) was used to assess for significant variance across the three groups, with age, sex, BMI, and IDS‐SR included as covariates. The *F* statistic and corresponding *p* value are reported from each ANCOVA. Subsequent pairwise comparisons were performed for outcomes with a significant ANCOVA. These pairwise comparisons were also adjusted for the same covariates as in the ANCOVA, with a Tukey‐corrected *p* value presented from these analyses.

All statistical analyses were performed in R Studio Statistical Software (v4.4.0; R Core Team 2024) (R Core Team 2024), while EEG and SWA processing was conducted in MATLAB (R2024a; v24.1.0; The MathWorks Inc., Natick, MA, USA) (The MathWorks Inc. 2024). An α of *p* < 0.05 was set for the threshold of significance across all analyses, with *p* < 0.10 indicating trend level significance.

## 3 Results

### 3.1 Sample Characteristics

Table 1 presents the characteristics for the primary analytic sample which included a total of 66 participants (*N* = 37 HD vs. *N* = 29 HSC). Participants were young to middle aged and predominantly female, white, and not Hispanic or Latino. Groups were statistically comparable in age, sex, race, and ethnicity, with average BMI higher in HD, relative to HSC (*p* = 0.006), though neither group had an average BMI in the overweight/obese range. HD also had significantly greater depressive symptomatology (*p* < 0.0001) relative to HSC.

**TABLE 1:** Sample characteristics.

Characteristic	HD	HSC	*p*
Sample Size (*N*)	37	29	—
Age (years)	26.9 ± 7.03	26.6 ± 5.60	0.85
Sex			
Female (%)	81.1	75.9	0.83
Male (%)	18.9	24.1	
Race (%)			
White	89.2	79.3	0.08
Asian	0.00	13.8	
Black or African American	5.4	6.9	
Not Reported	5.4	0.00	
Ethnicity (%)			
Hispanic or Latino	0.00	10.3	0.13
Not Hispanic or Latino	97.3	86.2	
Not Reported	2.7	3.5	
BMI (kg/m^2^)	24.6 ± 3.84	22.2 ± 2.9	**0.006**
IDS‐SR	25.3 ± 8.95	9.86 ± 6.80	**< 0.0001**

*Note:* Demographic, anthropomorphic, and depression data between hypersomnolence disorder (HD) and healthy sleeper controls (HSC). Body mass index (BMI); inventory of depressive symptomatology self‐report (ISD‐SR). Values are presented as means ± standard deviation, percentage of sample, or count. The *p* value from the statistical comparisons is provided, where appropriate. Statistically significant *p* values (< 0.05) are bolded.

### 3.2 Hypersomnolence Measures (Self‐Report and Objective) and Polysomnography

Table 2 presents the results from the primary analyses across hypersomnolence measures and polysomnography.

**TABLE 2:** Hypersomnolence measures and polysomnography.

Measure	HD *M* ± SD	HSC *M* ± SD	Unadjusted			Adjusted		
			*β* (SE)	*p*	$\eta_{p}^{2}$	*β* (SE)	*p*	$\eta_{p}^{2}$
Self‐report hypersomnolence measures								
ESS	13.9 ± 4.72	4.66 ± 2.55	9.21 (0.98)	**< 0.0001**	0.586	8.91 (1.43)	**< 0.0001**	0.398
SIQ	68.3 ± 16.6	32.3 ± 10.6	36.0 (3.56)	**< 0.0001**	0.623	22.1 (4.21)	**< 0.0001**	0.323
HSI – Total	21.9 ± 4.87	4.59 ± 2.99	17.4 (1.04)	**< 0.0001**	0.819	13.9 (1.36)	**< 0.0001**	0.642
HSI—Symptoms	10.3 ± 3.07	2.38 ± 1.50	7.96 (0.62)	**< 0.0001**	0.724	6.73 (0.86)	**< 0.0001**	0.512
FOSQ – 10	23.3 ± 5.08	31.8 ± 9.09	−8.51 (1.80)	**< 0.0001**	0.265	−5.33 (2.65)	**0.049**	0.065
Objective hypersomnolence measures								
MSLT MSL (min)	13.0 ± 5.43	15.9 ± 3.25	−2.90 (1.14)	**0.01**	0.092	−1.41 (1.71)	0.41	0.011
PVT – medRT (ms)	277 ± 52.0	250 ± 26.2	27.3 (10.6)	**0.01**	0.094	30.7 (15.4)	0.050	0.064
PVT – Lapses (*N*)	3.23 ± 2.68	1.88 ± 1.06	1.35 (0.53)	**0.01**	0.092	0.97 (0.78)	0.22	0.025
PST PUI	8.24 ± 3.33	7.61 ± 2.47	0.64 (0.74)	0.39	0.011	0.85 (1.07)	0.43	0.011
Polysomnography (PSG)								
TIB (min)	581 ± 73.8	548 ± 59.8	33.1 (16.9)	0.054	0.057	58.5 (23.5)	**0.02**	0.095
TST (min)	512 ± 73.2	454 ± 85.8	57.7 (19.6)	**0.005**	0.119	82.5 (28.9)	**0.006**	0.121
SE (%)	88.3 ± 7.40	82.6 ± 10.9	5.67 (2.26)	**0.01**	0.089	6.41 (3.46)	0.07	0.055
SOL (min)	16.2 ± 10.9	18.6 ± 25.8	−2.42 (4.70)	0.61	0.004	1.57 (7.03)	0.82	0.001
WASO (min)	52.4 ± 40.7	72.2 ± 46.0	−19.8 (10.7)	0.07	0.051	−22.8 (16.4)	0.17	0.032
Awakenings (*N*)	17.7 ± 9.02	19.2 ± 8.17	−1.50 (2.15)	0.49	0.008	2.02 (3.21)	0.53	0.007
REM Latency (min)	113 ± 58.5	123 ± 62.8	−9.75 (15.0)	0.52	0.007	−5.58 (22.2)	0.80	0.001
REM Duration (min)	132 ± 43.0	106 ± 35.9	25.7 (9.93)	**0.01**	0.095	36.0 (15.1)	**0.02**	0.088
REM Percentage (%)	25.6 ± 6.54	23.0 ± 5.48	2.54 (1.51)	0.10	0.042	3.57 (2.27)	0.12	0.040
N1 Duration (min)	16.5 ± 10.7	28.3 ± 13.5	−11.8 (2.97)	**0.0002**	0.198	−6.46 (4.44)	0.15	0.035
N1 Percentage (%)	3.33 ± 2.32	6.46 ± 3.68	−3.13 (0.74)	**< 0.0001**	0.217	−2.28 (1.12)	**0.047**	0.065
N2 Duration (min)	279 ± 63.4	205 ± 51.3	74.7 (14.5)	**< 0.0001**	0.294	85.4 (21.5)	**0.0002**	0.211
N2 Percentage (%)	54.5 ± 9.55	45.2 ± 8.73	9.24 (2.28)	**0.0001**	0.204	8.85 (3.47)	**0.01**	0.099
N3 Duration (min)	84.1 ± 32.9	115 ± 36.0	−31.0 (8.51)	**0.0005**	0.172	−32.5 (12.9)	**0.01**	0.096
N3 Percentage (%)	16.6 ± 6.77	25.3 ± 6.62	−8.66 (1.66)	**< 0.0001**	0.298	−10.1 (2.52)	**0.0002**	0.215
Arousals (*N*)	69.2 ± 29.8	47.6 ± 28.2	21.6 (7.30)	**0.004**	0.122	19.9 (11.2)	0.08	0.052
ArI (N/h)	8.20 ± 3.64	6.34 ± 3.98	1.86 (0.95)	0.055	0.057	0.71 (1.45)	0.63	0.004
AHI (N/h)	1.26 ± 1.67	0.54 ± 0.62	0.72 (0.33)	**0.03**	0.069	0.36 (0.50)	0.48	0.009

*Note:* Comparisons between hypersomnolence disorder (HD) and healthy sleeper controls (HSC) across hypersomnolence measures (self‐report and objective) and polysomnography (PSG). Self‐report hypersomnolence measures include Epworth sleepiness scale (ESS), sleep inertia questionnaire (SIQ), hypersomnia severity index – total score (HSI – Total), hypersomnia severity index – symptom specific score (HSI – Symptoms), and functional outcomes of sleepiness—10 item (FOSQ—10). Objective hypersomnolence measures include multiple sleep latency test—mean sleep latency (MSLT MSL; minutes), psychomotor vigilance task—median reaction time (PVT—medRT; ms), PVT—number of lapses with Tukey transformation (PVT—Lapses; number), and pupillographic sleepiness test—pupillary unrest index (PST—PUI). PSG outcomes include time in bed (TIB; minutes), total sleep time (TST; minutes), sleep efficiency (SE; %), wake after sleep onset (WASO; minutes), number of awakenings (Awakenings; number), rapid eye movement (REM) sleep latency (minutes), duration (minutes), and percentage (%), non‐rapid eye movement stage 1 (N1), stage 2 (N2), and stage 3 (N3) duration (minutes) and percentage (%), number of arousals (Arousals; number), arousal index (ArI; number per hour), and apnea‐hypopnea index (AHI; number per hour). Values are provided as means ± standard deviation, where appropriate. The regression coefficient (*β*), standard error (SE), *p* value and partial eta‐squared ($\eta_{p}^{2}$) are presented from the unadjusted and adjusted linear regressions, with the adjusted regressions accounting for age, sex, body mass index, and depressive symptom severity. Statistically significant *p* values (< 0.05) are bolded, and the adjusted analyses are shaded in light gray.

Consistent with group diagnoses, HD reported significantly worse daytime sleepiness, sleep inertia, hypersomnia symptom severity, and functional impairment due to sleepiness on all self‐report measures in both unadjusted and adjusted analyses compared to HSC. HD displayed significantly greater daytime sleep propensity on the MSLT (MSLTMSL *β* = −2.90; *p* = 0.01) in unadjusted analyses, that was no longer significant in adjusted models. HD demonstrated worse vigilance on the PVT, relative to HSC, evidenced by significantly slower reaction time (PVT—medRT *β* = 27.3; *p* = 0.01) and more lapses (PVT—Lapses *β* = 1.35; *p* = 0.01) in unadjusted analyses. A statistical trend was observed for PVT reaction time in the adjusted model (*p* = 0.050), while the difference in PVT lapses was neither significant nor of trend level significance in the adjusted analysis.

Groups differed significantly across several polysomnography variables. In both unadjusted and adjusted analyses, HD demonstrated significantly longer total sleep time (TST), less N1 percentage (% of TST), more N2 duration and percentage, less N3 duration and percentage, and greater REM duration. Significant differences were observed in unadjusted analyses for greater sleep efficiency and more arousals (number), with trend level significance observed for these relationships in adjusted analyses. HD also had a significantly increased AHI in unadjusted analyses, relative to HSC, yet averages for both groups were within the normal range for adults (AHI < 5), and no statistically significant difference was observed in the adjusted analysis. HD displayed longer time in bed that was of trend level significance in unadjusted analyses, with statistical significance observed for this relationship in adjusted analyses.

### 3.3 Normalized Slow Wave Activity (SWA)

Table 3 presents the results from the normalized SWA analyses for all‐night, cycle 1, cycle 2, and cycle 3 data.

**TABLE 3:** Normalized slow wave activity.

Channel	HD *M* ± SD	HSC *M* ± SD	Unadjusted			Adjusted		
			*β* (SE)	*p*	$\eta_{p}^{2}$	*β* (SE)	*p*	$\eta_{p}^{2}$
All‐Night								
F3	75.6 ± 5.55	79.5 ± 6.48	−3.92 (1.54)	**0.01**	0.099	−0.52 (2.17)	0.81	0.001
F4	76.9 ± 6.34	80.5 ± 6.17	−3.60 (1.55)	**0.02**	0.078	0.73 (2.14)	0.74	0.002
C3	75.2 ± 5.92	79.4 ± 5.51	−4.21 (1.42)	**0.004**	0.120	−1.24 (2.03)	0.54	0.006
C4	75.4 ± 5.84	80.3 ± 4.82	−4.86 (1.36)	**0.0007**	0.169	−2.18 (1.95)	0.27	0.021
O1	67.5 ± 5.93	71.1 ± 6.31	−3.62 (1.52)	**0.02**	0.082	−0.06 (2.19)	0.98	0.000
O2	68.2 ± 5.54	71.5 ± 6.19	−3.36 (1.48)	**0.03**	0.078	0.04 (2.07)	0.98	0.000
Cycle 1								
F3	81.9 ± 6.76	86.7 ± 4.30	−4.79 (1.50)	**0.002**	0.148	−6.36 (2.20)	**0.005**	0.134
F4	82.0 ± 7.55	86.5 ± 5.00	−4.51 (1.63)	**0.007**	0.107	−5.15 (2.46)	**0.04**	0.069
C3	80.8 ± 6.80	84.7 ± 5.22	−3.96 (1.53)	**0.01**	0.095	−5.61 (2.25)	**0.02**	0.095
C4	81.0 ± 6.88	85.7 ± 4.08	−4.74 (1.47)	**0.002**	0.142	−5.98 (2.17)	**0.008**	0.116
O1	73.8 ± 6.57	77.9 ± 5.83	−4.02 (1.56)	**0.01**	0.095	−6.20 (2.31)	**0.01**	0.110
O2	73.7 ± 6.83	78.0 ± 5.53	−4.35 (1.59)	**0.008**	0.109	−5.98 (2.33)	**0.01**	0.105
Cycle 2								
F3	75.2 ± 7.83	79.4 ± 7.70	−4.28 (2.00)	**0.04**	0.072	−0.66 (2.88)	0.82	0.001
F4	76.7 ± 8.05	80.1 ± 7.40	−3.42 (1.93)	0.08	0.047	0.16 (2.79)	0.95	0.000
C3	75.1 ± 8.02	78.6 ± 7.54	−3.53 (1.94)	0.07	0.049	−1.64 (2.90)	0.57	0.005
C4	75.3 ± 7.63	79.5 ± 6.99	−4.16 (1.84)	**0.03**	0.075	−2.19 (2.75)	0.43	0.011
O1	68.3 ± 7.68	71.4 ± 7.68	−3.06 (1.92)	0.12	0.039	−0.70 (2.88)	0.81	0.001
O2	67.9 ± 7.81	71.6 ± 7.53	−3.69 (1.95)	0.06	0.055	−0.83 (2.85)	0.77	0.002
Cycle 3								
F3	74.8 ± 7.95	75.5 ± 6.35	−0.76 (1.88)	0.69	0.003	1.10 (2.66)	0.68	0.003
F4	76.0 ± 8.23	75.7 ± 7.02	0.30 (1.92)	0.87	0.000	2.82 (2.80)	0.32	0.017
C3	74.2 ± 7.59	74.5 ± 6.81	−0.35 (1.80)	0.85	0.001	2.05 (2.66)	0.44	0.010
C4	74.8 ± 7.98	74.7 ± 6.90	0.13 (1.89)	0.95	0.000	2.53 (2.79)	0.37	0.014
O1	66.8 ± 7.97	67.0 ± 6.33	−0.16 (1.82)	0.93	0.000	3.81 (2.61)	0.15	0.036
O2	67.4 ± 7.61	67.3 ± 6.68	0.17 (1.83)	0.93	0.000	4.05 (2.61)	0.13	0.041

*Note:* Comparisons between hypersomnolence disorder (HD) and healthy sleeper controls (HSC) across normalized slow wave activity from the all‐night, cycle 1, cycle 2, and cycle 3 analyses. Group means ± standard deviations are provided for each electroencephalographic channel: F3, F4, C3, C4, O1, and O2. The regression coefficient (*β*), standard error (SE), *p* value and partial eta‐squared ($\eta_{p}^{2}$) are presented from the unadjusted and adjusted linear regressions, with the adjusted regressions accounting for age, sex, body mass index, and depressive symptom severity. Statistically significant *p* values (< 0.05) are bolded, and the adjusted analyses are shaded in light gray.

In all‐night analyses, HD displayed significantly reduced SWA across all channels, relative to HSC, with the effects largest in C4 (*β =* −4.86; *p* = 0.0007; $\eta_{p}^{2}$ = 0.169), followed by C3 (*β =* −4.21; *p* = 0.004; $\eta_{p}^{2}$ = 0.120). No significant differences were observed in the adjusted models for all‐night SWA.

Significantly reduced SWA was observed across all channels in unadjusted analyses for cycle 1, with the largest effects observed in F3 (*β =* −4.79; *p* = 0.002; $\eta_{p}^{2}$ = 0.148) and C4 (*β =* −4.74; *p* = 0.002; $\eta_{p}^{2}$ = 0.142). Statistically significant reductions were also observed across all channels in adjusted analyses.

In unadjusted analyses for cycle 2, significantly reduced SWA was observed in F3 and C4, with no statistically significant differences observed across adjusted analyses. No statistically significant differences were observed in either unadjusted or adjusted analyses for SWA in cycle 3.

### 3.4 Post Hoc Analyses: Comparisons Between HD/IH+, HD/IH−, and HSC

Post hoc analyses were performed that compared hypersomnolence disorder who met objective criteria for idiopathic hypersomnia (HD/IH+), those who did not (HD/IH−), and HSC. Of the 37 HD participants, 10 met criteria for the HD/IH+ group while the other 27 comprised the HD/IH− group. HD/IH+ participants displayed either PSG (≥ 660 min on ad libitum polysomnography) or MSLT (≤ 8 min) criterion for idiopathic hypersomnia, consistent with diagnostic criteria outlined in in the International Classification of Sleep Disorders, 3rd Edition, Text Revision, while HD/IH− participants did not meet either criterion (American Academy of Sleep Medicine 2023). Within the HD/IH+ group, eight participants met MSLT criterion without PSG criterion, one participant met PSG criterion without MSLT criterion, and one participant met both PSG and MSLT criteria.

Table S1 presents the post hoc analyses related to hypersomnolence measures and polysomnography. Significant variance across groups was observed for all self‐report and objective measures of hypersomnolence, except for PST PUI. Both HD/IH+ and HD/IH− reported significantly worse daytime sleepiness, sleep inertia, and hypersomnia symptoms, relative to HSC, while being statistically comparable to one another. HD/IH+ displayed significantly shorter MSLT MSL, relative to both HD/IH− and HSC, while HD/IH− and HSC were statistically comparable to one another. No significant pairwise differences were observed for the FOSQ‐10 or PVT outcomes.

Significant variance across groups was observed for polysomnography total sleep time (TST), sleep efficiency, wake after sleep onset, REM duration (minutes), N1, N2, and N3 duration and percentage (% TST), and arousals (number). Notably, both HD/IH+ and HD/IH− displayed significantly longer TST, relative to HSC, while being statistically comparable to one another. HD/IH+ had a significantly greater sleep efficiency and reduced wake after sleep onset, relative to both HD/IH− and HSC, while HD/IH− and HSC were statistically comparable to one another. Both HD/IH+ and HD/IH− had statistically less N3 percentage, relative to HSC, while being statistically comparable to one another.

Table 4 presents the post hoc analyses for all night, cycle 1, cycle 2, and cycle 3 SWA. Significant variance across groups was observed for all channels in both all‐night and cycle 1 SWA analyses. No statistically significant pairwise differences were observed in all‐night SWA analyses. In cycle 1 analyses, HD/IH− had significantly less normalized SWA in all channels except F4, relative to HSC, while being statistically comparable to HD/IH+ across all channels. HD/IH+ was statistically comparable to HSC for all channels in cycle 1. In cycle 2, significant variance across groups was observed in F4, C4, and O2, while no significant variance was observed in any channels across cycle 3 analyses. No pairwise group differences were observed in cycle 2 or 3.

**TABLE 4:** Post hoc analyses: normalized slow wave activity across HD/IH+, HD/IH−, and HSC.

Channel	Means ± standard deviations			ANCOVA		Adjusted pairwise comparisons (*p*)		
	HSC	HD/IH−	HD/IH+	*F*	*p*	HD/IH− vs. HSC	HD/IH+ vs. HSC	HD/IH− vs. HD/IH+
All‐Night								
F3	79.5 ± 6.48	76.3 ± 5.56	74.0 ± 5.46	3.61	**0.03**	0.97	0.90	0.92
F4	80.5 ± 6.17	78.3 ± 5.54	73.2 ± 7.09	6.09	**0.004**	0.92	0.64	0.26
C3	79.4 ± 5.51	76.1 ± 6.15	73.0 ± 4.84	5.75	**0.005**	0.83	0.65	0.80
C4	80.3 ± 4.82	75.9 ± 6.22	74.0 ± 4.65	7.05	**0.002**	0.52	0.67	0.99
O1	71.1 ± 6.31	68.6 ± 6.32	64.5 ± 3.54	4.73	**0.01**	1.00	0.76	0.61
O2	71.5 ± 6.19	69.4 ± 5.63	65.1 ± 4.08	5.00	**0.01**	1.00	0.74	0.56
Cycle 1								
F3	86.7 ± 4.30	81.2 ± 7.10	83.7 ± 5.81	5.64	**0.006**	**0.01**	0.39	0.64
F4	86.5 ± 5.00	81.9 ± 7.22	82.2 ± 8.79	3.77	**0.03**	0.10	0.40	0.98
C3	84.7 ± 5.22	79.9 ± 7.40	82.9 ± 4.44	4.56	**0.01**	**0.04**	0.68	0.44
C4	85.7 ± 4.08	80.0 ± 7.45	83.5 ± 4.37	6.92	**0.002**	**0.02**	0.73	0.24
O1	77.9 ± 5.83	73.6 ± 6.96	74.6 ± 5.72	3.71	**0.03**	**0.03**	0.23	0.96
O2	78.0 ± 5.53	72.9 ± 7.22	75.6 ± 5.61	4.86	**0.01**	**0.03**	0.62	0.50
Cycle 2								
F3	79.4 ± 7.70	76.2 ± 7.50	72.6 ± 8.38	2.92	0.06	—	—	—
F4	80.1 ± 7.40	78.6 ± 6.97	71.7 ± 8.97	5.04	**0.01**	0.99	0.10	0.31
C3	78.6 ± 7.54	76.3 ± 8.11	71.8 ± 7.13	2.85	0.07	—	—	—
C4	79.5 ± 6.99	76.4 ± 7.75	72.4 ± 6.86	3.48	**0.04**	0.73	0.56	0.40
O1	71.4 ± 7.68	69.6 ± 7.35	64.9 ± 7.85	2.61	0.08	—	—	—
O2	71.6 ± 7.53	69.4 ± 6.96	64.2 ± 8.89	3.55	**0.04**	0.97	0.42	0.51
Cycle 3								
F3	75.5 ± 6.35	76.0 ± 8.12	71.7 ± 6.95	1.74	0.18	—	—	—
F4	75.7 ± 7.02	77.4 ± 8.36	72.3 ± 6.94	2.07	0.14	—	—	—
C3	74.5 ± 6.81	75.7 ± 7.63	70.2 ± 6.17	2.18	0.12	—	—	—
C4	74.7 ± 6.90	75.9 ± 8.47	72.0 ± 5.98	1.02	0.37	—	—	—
O1	67.0 ± 6.33	68.2 ± 8.67	63.1 ± 4.18	2.05	0.14	—	—	—
O2	67.3 ± 6.68	69.2 ± 7.89	63.1 ± 4.81	2.92	0.06	—	—	—

*Note:* Post hoc comparisons between hypersomnolence disorder who met objective criteria for idiopathic hypersomnia (HD/IH+), those who did not (HD/IH−), and healthy sleeper controls (HSC) across normalized slow wave activity for all‐night, cycle 1, cycle 2, and cycle 3 analyses. Group means ± standard deviations are provided for each electroencephalographic channel: F3, F4, C3, C4, O1, and O2. The *F* statistic and *p* value are provided from the ANCOVA that adjusted for age, sex, body mass index, and depressive symptom severity. The Tukey corrected *p* values from the adjusted pairwise comparisons are provided, which included the same covariates as the ANCOVA. Statistically significant *p* values (< 0.05) are bolded, with the ANCOVA and adjusted pairwise comparisons shaded in light gray.

## 4 Discussion

This investigation was designed to further examine reductions in nocturnal sleep slow wave activity as a potential physiological signature of hypersomnolence disorder (HD) using a distinct dataset from prior investigations in this area of research previously reported from our laboratory (Plante et al. 2019). Analyses compared an unmedicated, clinical sample of individuals with HD against a group of healthy sleeper controls without sleep complaints (HSC). Using standard visual stage scoring, HD exhibited reduced N3 sleep, both in duration and percentage of total sleep time, compared to HSC, coarsely suggesting reductions in slow waves in the disorder. This finding parallels prior meta‐analytic evidence implicating decreased N3 in idiopathic hypersomnia (IH) (Plante 2018). Extending beyond visual stage metrics, our results also showed normalized slow wave activity (SWA) reductions in HD, particularly in the first cycle of non‐rapid eye movement (NREM) sleep. These findings replicate and build upon prior work from our group (Plante et al. 2019), further supporting the notion of SWA reductions in early NREM sleep as a physiological feature of HD. Post hoc analyses comparing HD participants who met objective criteria for IH (HD/IH+) to those who did not (HD/IH−) showed no significant differences in normalized SWA, suggesting a potential shared underlying finding among these related constructs. Taken together, the results from this investigation steer towards future research that examines the causal role of slow waves in the pathophysiology of HD and unexplained hypersomnolence, broadly. These findings also highlight the importance of further investigations aimed at clarifying both the shared and distinct physiological features of HD and IH.

Our primary power spectral analyses replicate reductions in normalized SWA as a potential electroencephalographic (EEG) signature of HD, aligning with previous findings from our laboratory in entirely separate cohorts of HD and controls (Plante et al. 2019). We have now repeatedly observed that these normalized SWA reductions are most pronounced during the first sleep cycle, and in central EEG channels. Importantly, this investigation extends our prior work conducted with high‐density, 256‐channel EEG to the more clinically common 6‐channel EEG montage, thereby increasing the generalizability and clinical relevance of these results. These findings also align with prior research demonstrating reductions in absolute SWA among persons with IH compared to controls, that occurs predominantly during the early portions of the sleep episode (Sforza et al. 2000). Overall, these findings broadly support the concept that slow wave activity, which is closely tied to the restorative aspects of sleep (Dijk 2009; Mander et al. 2010; Achermann et al. 2017), is reduced in HD, and may serve as a physiological signature of the disorder as well as unexplained hypersomnolence, more broadly.

The corroboration of SWA reductions in HD may carry specific importance for guiding future exploration of slow wave enhancement as a potential therapy for HD (Fehér et al. 2021). Nonpharmacological strategies, such as closed‐loop auditory stimulation (Esfahani et al. 2023) and neurostimulation approaches (e.g., transcranial electric and magnetic stimulation) (Grimaldi et al. 2020), have emerged as potential techniques for slow wave enhancement. Notably, oxybate formulations, which have been demonstrated to be an effective therapy for persons with idiopathic hypersomnia (Dauvilliers et al. 2022), also increase slow wave sleep and activity in both healthy controls and persons with sleep and psychiatric conditions (Lapierre et al. 1990; Van Cauter et al. 1997; Mamelak et al. 2004; Bavato et al. 2025). Given the accumulating evidence of SWA reductions in HD, investigating the effects of slow wave enhancement through pharmacologic and nonpharmacologic strategies may be a fruitful avenue for establishing a causal role of slow waves in the pathophysiology of HD. Importantly, some neurostimulation techniques may also allow for targeted, regionally precise slow wave enhancement (Ruch et al. 2022), which could further elucidate the specific brain structures involved in the disorder.

Although this study was not primarily designed to examine differences in SWA between HD and IH, we utilized post hoc analyses to explore SWA differences between HD subtypes, those who met diagnostic criteria for IH (HD/IH+) and those who did not (HD/IH−). These analyses did not demonstrate that HD/IH+ participants had significantly reduced SWA compared to HSC or HD/IH− participants. While these findings must be interpreted with great caution given limited sample size in the HD/IH+ group, it is notable that mean SWA values in this group were descriptively below HSC but above HD/IH‐ group. In general, persons meeting objective PSG/MSLT criteria for IH are assumed to have a more severe condition than those who do not demonstrate these findings. Under this presupposition, reduced SWA, particularly in cycle 1, if truly a maker for unexplained hypersomnolence, would be more greatly reduced in HD/IH+ compared to reductions in HD/IH−, which is not what was observed in this study. One possible explanation is that reduced SWA is a more relevant biomarker in the subgroup of persons with unexplained hypersomnolence who do not meet objective criteria for IH, and thus SWA in cycle 1 may be a relevant adjunctive measure in this subgroup, potentially complementing and informing existing diagnostic criteria. An alternative explanation is that the objective measures currently required to diagnose IH are not related to reduced SWA as a relevant electrophysiological finding in unexplained hypersomnolence, which would be supported by the limited reliability of the MSLT in IH (Trotti et al. 2013; Lopez et al. 2017). Future adequately powered studies that evaluate the relevance of objective PSG/MSLT findings in subtypes of HD and IH in relation to sleep slow waves are clearly indicated.

## 5 Limitations

There are several limitations of this investigation that merit consideration. First, the demographic characteristics of the sample, including being predominantly female, non‐Hispanic or Latino, and white, limit the generalizability of the findings. The female predominance also precluded evaluation of potential sex‐related interactions in slow wave activity. Future studies in more demographically diverse cohorts, including more balanced sex distributions, are needed, although it is worth noting that unexplained hypersomnolence is more commonly observed in women, as reflected in this investigation's sample (Ali et al. 2009; Mallampalli and Carter 2014). Second, despite the sample being unmedicated and well‐characterized, the overall sample size was modest. Replication in larger cohorts of patients with unexplained hypersomnolence that includes balanced representation of HD/IH+ and HD/IH− phenotypes will strengthen the confidence in these findings and help clarify potential physiological distinctions and similarities between HD and IH. Third, as clinical participants were utilized for the HD group, an acclimation night to the sleep laboratory was not employed, which was also the case for HSC; however, the potential of a first‐night effect differentially affecting HD and HSC cannot be ruled out. Fourth, actigraphy data were not universally available for clinically referred patients, limiting our ability to examine the effects of habitual sleep duration on slow wave activity with sufficient accuracy. Despite participants being asked not to nap on the day prior to the overnight PSG, it is possible that some participants had brief or unscheduled naps which could have affected homeostatistic sleep pressure and, consequentially, SWA. Lastly, while the use of a 6‐channel EEG montage aligns with standard clinical practice and enhances generalizability, it limits the spatial resolution of EEG analyses and inhibits the application other advanced EEG methods such as source localization. Future work utilizing high‐density EEG and complementary methods will be important for advancing the understanding of the neurophysiology underlying HD, IH, and unexplained hypersomnolence, more broadly.

## 6 Conclusion

In summary, this investigation provides further evidence that reductions in normalized slow wave activity (SWA), particularly in the first cycle of NREM sleep, may serve as a physiological signature of hypersomnolence disorder (HD). These results replicate previous work of reduced N3 sleep and SWA in HD, strengthening the notion that slow wave deficiencies characterize HD. The lack of observed differences in post hoc analyses that explored SWA between participants with HD who met diagnostic criteria for idiopathic hypersomnia (HD/IH+) and those who did not (HD/IH‐) raises the possibility of shared underlying physiological features between HD and idiopathic hypersomnia (IH). However, the small sample sizes of these HD subgroups, particularly the HD/IH+ group, warrant cautious interpretation and underscores the need for future research in larger, more balanced samples to clarify the shared and distinct physiological features between HD and IH. Collectively, these findings contribute to the growing body of evidence implicating reductions in nocturnal slow wave sleep and activity as a signature of unexplained hypersomnolence, guiding future research to examine the causal role of slow waves in the pathophysiology of these disorders.

## Author Contributions

**Jesse D. Cook:** writing – original draft (equal); conceptualization (equal); visualization (lead); methodology (lead); formal analysis (lead). **Brady A. Riedner:** writing – review and editing (equal); methodology (supporting); formal analysis (supporting); resources (supporting). **Meredith E. Rumble:** writing – review and editing (equal); conceptualization (equal); supervision (supporting). **Michael R. Goldstein:** writing – review and editing (equal); methodology (supporting); formal analysis (supporting); resources (supporting). **Ana Maria Vascan:** writing – review and editing (equal); project administration (equal). **Kieulinh M. Tran:** writing – review and editing (equal); project administration (equal). **David T. Plante:** writing – original draft (equal); conceptualization (equal); visualization (supporting); methodology (supporting); formal analysis (supporting); funding acquisition (lead); supervision (lead).

## Funding

This study was supported by grants from the American Sleep Medicine Foundation (138‐SR‐16) and National Institute of Nursing Research (R21NR018288).

## Ethics Statement

All participants provided informed consent and investigation oversight was provided by the UW‐Madison Health Sciences Institutional Review Board.

## Conflicts of Interest

Cook previously served as a consultant to Somni, Cerno Health, and Bodymatter Inc., with these financially compensated affiliations unrelated to the current study. Rumble receives grant support from Merck, unrelated to the current study. Goldstein has received grant support from the National Science Foundation, Mind & Life Institute, National Institutes of Health (Harvard Sleep Research T32), and Osher Center for Integrative Medicine at Harvard Medical School; and has served as a consultant for Bodymatter Inc., Harvard Football Players Study, and Cerebra Health Inc., unrelated to the current study. Plante has received grant support from NIA, NINR, NIMH, American Sleep Medicine Foundation, the Brian and Behaviour Research Foundation, Wisconsin Alumni Research Foundation, Alzheimer's Association, and the University of Illinois at Chicago Occupational and Environmental Health and Safety Education and Research Center/National Institute for Occupational Safety and Health; and has served as a consultant and/or advisory board member for Teva Australia, Harmony Biosciences, Takeda, Centesa, Apnimed, Aditium Bio LLC, Jazz Pharmaceuticals and Alkermes, unrelated to the current study.

## Supporting information


**Table S1:** Post Hoc: Hypersomnolence Measures and Polysomnography Across HD/IH+, HD/IH−, and HSC.

## Data Availability

The data used in this investigation can be made available upon reasonable request to the corresponding author.
