# Supplementary material for: Slow Wave Activity During Nocturnal Sleep in Hypersomnolence Disorder
Source: J Sleep Res. 2026 May 29;35(5):e70353. doi: 10.1111/jsr.70353 (PMC13619009; doi:10.1111/jsr.70353)
Supplement: Supplementary file 1 [file JSR-35-e70353-s001.docx]

**Supplementary Table 1**

*Post Hoc: Hypersomnolence Measures and Polysomnography Across HD/IH+, HD/IH-, and HSC*

| **Measure** | **HSC**  *M ± SD* | **HD/IH-**  *M ± SD* | **HD/IH+**  *M ± SD* | **ANCOVA** | | **Adjusted Pairwise Comparisons (*p* value)** | | |
| --- | --- | --- | --- | --- | --- | --- | --- | --- |
|  |  |  |  | ***F*** | ***p value*** | **HD/IH- vs. HSC** | **HD/IH+ vs. HSC** | **HD/IH- vs. HD/IH+** |
|  |  |  |  |  |  |  |  |  |
| ***Self-Report Hypersomnolence Measures*** | | |  |  |  |  |  |  |
| ESS | 4.66 ± 2.55 | 13.0 ± 4.95 | 16.1 ± 3.31 | 50.1 | **< 0.0001** | **< 0.0001** | **< 0.0001** | 0.09 |
| SIQ | 32.3 ± 10.6 | 65.4 ± 16.2 | 75.8 ± 15.9 | 80.8 | **< 0.0001** | **< 0.0001** | **0.0004** | 0.79 |
| HSI – Total | 4.59 ± 2.99 | 20.9 ± 4.66 | 24.9 ± 4.48 | 191 | **< 0.0001** | **< 0.0001** | **< 0.0001** | 0.18 |
| HSI – Symptoms | 2.38 ± 1.50 | 10.0 ± 3.07 | 11.3 ± 3.00 | 94.9 | **< 0.0001** | **< 0.0001** | **< 0.0001** | 0.65 |
| FOSQ – 10 | 31.8 ± 9.09 | 24.2 ± 5.21 | 20.8 ± 3.93 | 12.1 | **< 0.0001** | 0.13 | 0.15 | 0.77 |
|  |  |  |  |  |  |  |  |  |
| ***Objective Hypersomnolence Measures*** | | |  |  |  |  |  |  |
| MSLT MSL | 15.9 ± 3.25 | 15.7 ± 2.97 | 5.71 ± 3.47 | 42.8 | **< 0.0001** | 0.64 | **< 0.0001** | **< 0.0001** |
| PVT – medRT | 250 ± 26.2 | 276 ± 55.7 | 281 ± 43.2 | 3.71 | **0.03** | 0.13 | 0.23 | 0.92 |
| PVT – Lapses | 1.88 ± 1.06 | 3.07 ± 2.83 | 3.68 ± 2.3 | 3.56 | **0.03** | 0.46 | 0.43 | 0.85 |
| PST PUI | 7.61 ± 2.47 | 7.89 ± 2.79 | 9.19 ± 4.53 | 1.10 | 0.34 | ----- | ----- | ----- |
|  |  |  |  |  |  |  |  |  |
| ***Polysomnography (PSG)*** | |  |  |  |  |  |  |  |
| TIB | 548 ± 59.8 | 582 ± 59.3 | 579 ± 108 | 2.56 | 0.09 | ----- | ----- | ----- |
| TST | 454 ± 85.8 | 500 ± 60.6 | 544 ± 96.1 | 5.91 | **0.005** | **0.02** | **0.003** | 0.12 |
| SE | 82.6 ± 10.9 | 86.1 ± 7.38 | 94.2 ± 2.97 | 6.27 | **0.003** | 0.16 | **0.003** | **0.02** |
| SOL | 18.6 ± 25.8 | 19.3 ± 10.8 | 7.90 ± 5.87 | 1.55 | 0.22 | ----- | ----- | ----- |
| WASO | 72.2 ± 46.0 | 62.1 ± 42.7 | 26.3 ± 18.1 | 4.34 | **0.02** | 0.37 | **0.01** | **0.03** |
| Awakenings | 19.2 ± 8.17 | 18.0 ± 7.63 | 17.0 ± 12.5 | 0.24 | 0.79 | ----- | ----- | ----- |
| REM Latency | 123 ± 62.8 | 119 ± 60.2 | 97.2 ± 53.6 | 0.69 | 0.51 | ----- | ----- | ----- |
| REM Duration | 106 ± 35.9 | 127 ± 41.1 | 146 ± 47.0 | 4.20 | **0.02** | 0.06 | **0.02** | 0.28 |
| REM Percentage | 23.0 ± 5.48 | 25.1 ± 6.77 | 26.7 ± 6.06 | 1.68 | 0.19 | ----- | ----- | ----- |
| N1 Duration | 28.3 ± 13.5 | 17.8 ± 11.8 | 13.2 ± 5.95 | 7.85 | **0.001** | 0.33 | 0.36 | 0.86 |
| N1 Percentage | 6.46 ± 3.68 | 3.64 ± 2.58 | 2.47 ± 1.14 | 8.72 | **0.0005** | 0.12 | 0.13 | 0.72 |
| N2 Duration | 205 ± 51.3 | 272 ± 63.2 | 299 ± 63.0 | 14.3 | **< 0.0001** | **0.0006** | **0.0008** | 0.29 |
| N2 Percentage | 45.2 ± 8.73 | 54.3 ± 10.2 | 55.0 ± 8.00 | 7.54 | **0.001** | **0.04** | 0.14 | 0.97 |
| N3 Duration | 115 ± 36.0 | 83.1 ± 31.2 | 86.6 ± 38.8 | 6.28 | **0.003** | **0.04** | 0.28 | 0.97 |
| N3 Percentage | 25.3 ± 6.62 | 17.0 ± 6.95 | 15.8 ± 6.53 | 13.2 | **< 0.0001** | **0.0006** | **0.005** | 0.77 |
| Arousals | 47.6 ± 28.2 | 68.3 ± 30.4 | 71.9 ± 29.5 | 4.23 | **0.02** | 0.19 | 0.36 | 0.98 |
| ArI | 6.34 ± 3.98 | 8.29 ± 3.88 | 7.97 ± 3.10 | 1.86 | 0.17 | ----- | ----- | ----- |
| AHI | 0.54 ± 0.62 | 1.33 ± 1.88 | 1.06 ± 0.96 | 2.38 | 0.10 | **-----** | **-----** | **-----** |
|  |  |  |  |  |  |  |  |  |

**Supplementary Table 1.** *Post hoc* comparisons between hypersomnolence disorder who met objective criteria for idiopathic hypersomnia (HD/IH+), those who did not (HD/IH-), and healthy sleeper controls (HSC) across hypersomnolence measures and polysomnography. Group means ± standard deviation (*M* ± *SD*) are provided for each EEG channel: F3, F4, C3, C4, O1, and O2. Self-report hypersomnolence measures include Epworth sleepiness scale (ESS), sleep inertia questionnaire (SIQ), hypersomnia severity index – total score (HSI – Total), hypersomnia severity index – symptom specific score (HSI – Symptoms), and functional outcomes of sleepiness – 10 item (FOSQ – 10). Objective hypersomnolence measures include multiple sleep latency test – mean sleep latency (MSLT MSL; minutes), psychomotor vigilance task – median reaction time (PVT – medRT; ms), PVT – number of lapses with Tukey transformation (PVT – Lapses; number), and pupillographic sleepiness test – pupillary unrest index (PST – PUI). PSG outcomes include time in bed (TIB; minutes), total sleep time (TST; minutes), sleep efficiency (SE; %), wake after sleep onset (WASO; minutes), number of awakenings (Awakenings; number), rapid eye movement (REM) sleep latency (minutes), duration (minutes), and percentage (%), non-rapid eye movement stage 1 (N1), stage 2 (N2), and stage 3 (N3) duration (minutes) and percentage (%), number of arousals (Arousals; number), arousal index (ArI; number per hour), and apnea-hypopnea index (AHI; number per hour). The *F* statistic and *p* value are provided from the ANCOVA that adjusted for age, sex, body mass index, and depressive symptom severity. For outcomes with a significant ANCOVA, the Tukey corrected *p* values from the adjusted pairwise comparisons are provided. Statistically significant *p* values (< 0.05) are bolded.
